# Development and Validation of a Protocol for Pregnant Women Based on the Brazilian Dietary Guidelines

**DOI:** 10.1055/s-0042-1756213

**Published:** 2022-12-29

**Authors:** Cláudia Raulino Tramontt, Juliana Giaj Levra de Jesus, Thanise Sabrina Souza Santos, Fernanda Rauber, Maria Laura da Costa Louzada, Vanessa Del Castillo Couto, Jacqueline Resende Berriel Hochberg, Patrícia Constante Jaime

**Affiliations:** 1 Núcleo de Pesquisas Epidemiológicas em Nutrição e Saúde da Universidade de São Paulo, São Paulo, SP, Brazil; 2 Universidade Federal de Minas Gerais, Belo Horizonte, MG, Brazil; 3 Faculdade de Medicina, Universidade de São Paulo, SP, Brazil

**Keywords:** dietary guidelines, practice guidelines, prenatal nutrition, primary health care, validation study, guias alimentares, guias de prática clínica, nutrição pré-natal, atenção primária à saúde, estudo de validação

## Abstract

**Objective** To develop and validate a protocol for the use of the Dietary Guidelines for the Brazilian Population (DGBP) in the individual dietary advice for pregnant women assisted in primary healthcare (PHC).

**Methods** Methodological study that involved the elaboration of a protocol in six steps: definition of the format, definition of the instrument to evaluate food consumption, systematization of evidence on food and nutrition needs of pregnant women, extraction of DGBP recommendations, development of messages of dietary guidelines and content, and face validity. The analyses of the validation steps were carried out by calculating the Content Validity Index (CVI) and thematic content analysis.

**Results** As products of the steps, the protocol structure was defined and the dietary advice for pregnant women were elaborated, considering physiological changes, food consumption, nutritional and health needs, and socioeconomic conditions of this population. The protocol was well evaluated by experts and health professionals in terms of clarity, relevance (CVI > 0.8), and applicability. In addition, the participants made some suggestions to improve the clarity of the messages and to expand the applicability of the instrument with Brazilian pregnant women.

**Conclusion** The instrument developed fills a gap in clinical protocols on dietary advice for pregnant women focused on promoting a healthy diet, contributing to a healthy pregnancy. In addition, it demonstrates potential to contribute to the qualification of PHC professionals and to the implementation of the DGBP recommendations.

## Resumo

**Objetivo** Desenvolver e validar um protocolo para uso do Guia Alimentar para a População Brasileira (GAPB) na orientação alimentar individual de gestantes atendidas na atenção primária à saúde (APS).

**Métodos** Estudo metodológico que envolveu a elaboração de um protocolo em seis etapas: definição do formato do documento, definição do instrumento de avaliação do consumo alimentar, sistematização de evidências sobre necessidades de alimentação e nutrição de gestantes, extração das recomendações do GAPB, desenvolvimento de mensagens de orientação alimentar e validação de conteúdo e validação aparente. As análises das etapas de validação foram realizadas através do cálculo do índice de validade de conteúdo e análise temática de conteúdo.

**Resultados** Como produtos das etapas, a estrutura do protocolo foi definida e as orientações alimentares de gestantes foram elaboradas, considerando as alterações fisiológicas, consumo alimentar, necessidades nutricionais e de saúde e condições socioeconômicas desta população. O protocolo foi bem avaliado por especialistas e profissionais de saúde nos critérios de clareza, pertinência (índice de validade de conteúdo > 0,8) e aplicabilidade. Além disso, os participantes deram sugestões para melhoria da clareza das mensagens e para ampliar a aplicabilidade do instrumento em gestantes brasileiras.

**Conclusão** O instrumento desenvolvido preenche uma lacuna sobre protocolos clínicos de orientação alimentar para gestantes focado na promoção da alimentação saudável, contribuindo para uma gestação saudável. Além disso, o instrumento demonstra potencial para contribuir na qualificação de profissionais da APS e implementação das recomendações do GAPB.

## Introduction

Food and nutrition have been widely discussed in the global public policy agenda due to their importance in human health. 1 2 During pregnancy, a healthy diet favors good fetal development and women's health and well-being, in addition to preventing the onset of diseases such as gestational diabetes, hypertension, and excessive weight gain, and is associated with better health conditions in childhood and adult life. 3 4 For this reason, many countries have invested in the development of advices, recommendations, and guidelines to support adequate nutrition to ensure healthy pregnancy and delivery. 5 6 7 8

Pregnancy can be a window of opportunity for dietary interventions, since pregnant women tend to change their diet due to their new condition and in favor of the fetus health. However, this change does not always occur in the sense of improving the diet. Recent data on the diet of Brazilian pregnant women show a high consumption of ultraprocessed foods and a low-quality diet, evidenced by the low consumption of fruits and vegetables, especially in pregnant teens, mulatto and black women, and smokers. 9 10 11 12 13

The monitoring carried out by health professionals during prenatal care is a good opportunity to provide pregnant women with adequate advice on nutrition. However, dietary advice in prenatal care does not always occur, and when it does occur, it is carried out inappropriately or limited in content, not always consistent with current dietary guidelines, insufficient to meet the health needs of pregnant women, in addition to occurring from a vertical relationship between professional and user, perpetuating the care discontinuity, and nonadherence to dietary advice. 14 15 16 17 18

The Dietary Guidelines for the Brazilian Population (DGBP) contains healthy diet recommendations officially adopted by the Ministry of Health throughout the national territory which are applicable to the entire population > 2 years old. 19 However, which recommendations on healthy diets and how they should be directed to pregnant women have not yet been established.

Clinical practice protocols are considered the “basis of accurate advising” and the foundation of evidence-based practice. 20 The absence of instruments such as these to guide the clinical practice of health professionals is a limiting factor for adequate dietary advice to occur. A systematic review on the quality of clinical practice guidelines for nutrition during pregnancy found most of the guidelines inadequate in terms of development rigor, content of nutritional recommendations, applicability, and the declaration of funding sources and conflict of interests. 21 These findings highlight the lack of tools for dietary advice during pregnancy that help clinical practice, are updated based on the best available scientific evidence, improve prognosis and qualify the health care.

Considering the importance of adequate dietary advice in the gestational period and the absence of instruments to support clinical practice, the present study aimed to develop and validate a protocol for using the DGBP in dietary advice for pregnant women assisted at primary healthcare (PHC).

## Methods

This is a methodological study on the development and validation of a protocol for the use of the DGBP for dietary advice for pregnant women assisted at PHC.

### The Dietary Guidelines for the Brazilian Population

Published in 2014, the DGBP is an official document that presents recommendations on healthy eating based on evidence on the relationship between diet and health, systematized in 5 chapters. Its elaboration derived from a holistic perspective, comprising the biological, cultural, social, and environmental dimensions of food. This document is an instrument to support nutrition education actions at the Brazilian Unified Health System (SUS, in the Portuguese acronym). 19 The development of this protocol is part of a matrix project that assumed the DGBP as the technical reference for guidance and promotion of healthy eating. The project aimed to develop a series of individual dietary protocols for applying the DGBP in different life cycles/events, aimed at health promotion and care qualification in relation to the most prevalent dietary needs in the PHC context. The methodological basis that guided the development of the series was described in a previous publication. 22

### Development of the Protocol to Use the Brazilian Dietary Guidelines on Dietary Advice for Pregnant Women

To elaborate the series of protocols, a team of eight researchers free of conflict of interests and experts in dietary advice based on the DGBP and on PHC was formed. The elaboration of the protocol to use the DGBP for pregnant women was carried out following six steps. The first addressed the definition of the protocol format whereas the second dealt with the definition of the instrument for assessing food consumption; the third step consisted of extracting the recommendations from the DGBP applicable to individual dietary advice; the fourth was the evidence systematization on the dietary and nutrition needs of pregnant women; the fifth addressed the development of dietary advice messages for pregnant women; and, finally, in the last step, the content and face validity was carried out.

#### Step 1. Format Definition

At this step, documents on the elaboration of guidelines for clinical practice were analyzed, as well as PHC protocols already published in Brazil. The objective was to investigate the possible formats, analyze their characteristics and identify the most appropriate ones to guide dietary advice for promoting healthy eating in individual appointments in PHC.

#### Step 2. Definition of the Instrument to Assess Food Consumption

To support decision-making for dietary advice, a search was carried out on existing instruments for assessing food consumption. The objective was to identify an assessment instrument of the main recommendations from the DGBP that could be used by any PHC professional during individual appointments.

#### Step 3. Evidence Systematization on Food and Nutrition Needs of Pregnant Women

To identify the scientific evidence on the dietary needs and specificities and food consumption of pregnant women, a literature review was carried out in the Scielo, Lilacs, PubMed, and gray literature databases (academic Google). We searched for original articles published in English, Portuguese, or Spanish with evidence from national surveys. The search was conducted using the terms *food consumption* OR *food intake* AND *pregnant woman* OR *pregnancy* OR *prenatal nutrition* AND *brazil* . Additionally, analyses were carried out on the 2017–2018 Family Budget Survey 23 with a focus on pregnant women and searches in books and in technical materials that included dietary advice for pregnant women.

#### Step 4. Extracting the Recommendations

Two researchers carried out a systematic reading of chapters 2 (Food choice), 3 (From food to meals), 4 (Ways of eating), and 5 (Understanding and overcoming obstacles) of the DGBP in order to identify appropriate and relevant recommendations that were in line with the indicators of the food consumption assessment instrument identified in Step 2 (Definition of the instrument). Furthermore, additional recommendations considered relevant to be included in the nutritional advice for pregnant women were identified, even if they were not addressed by the food consumption assessment instrument. The other five researchers in the expert team reviewed the recommendations to elaborate the final list.

#### Step 5. Development of dietary advice messages for pregnant women

At this step, dietary advice messages were prepared based on the recommendations of the DGBP extracted in Step 3 (Evidence systematization) considering the specificity of the diet of pregnant women systematized in Step 4 (Extraction of recommendations), also including the obstacles to a healthy diet faced by this population.

#### Step 6. Content and Face Validity

As a product of the previous steps, version 1 of the protocol was elaborated. This first version was sequentially submitted to content and face evaluations.

6.1–Content validity: 19 experts in the areas of health and nutrition for pregnant women, dietary guideline, and nutritional care in PHC were invited to compose a panel of judges. The experts received by email the first version of the protocol, support materials explaining the validation process, the systematization of life cycle evidence (result of Step 4), and an online form to assess the clarity and relevance of each protocol component using a 4-point scale (1–the item is not clear/relevant; 2–major revisions are needed to make the item clear/relevant; 3–minor revisions are necessary to make the item clear/relevant; and, 4–the item is clear/relevant). For each item judged as not clear/relevant, a justification was requested. The form also had a field for additional suggestions. The protocol components are identified in Table 1 . After completing the form, the experts were invited to participate in an online focus group to collect general impressions about the protocol. Upon the collaboration from the experts, changes were made to version 1 of the protocol in order to contemplate the pertinent suggestions, resulting in version 2.

**Table 1:** Description of the content validity index by the protocol component of the protocols for the Dietary Guidelines for the Brazilian Population use in nutrition advising for pregnant women

	Content Validity Index		
Component	Clarity (min-max)	Relevance (min-max)	Average CVI
Introductory text	1 (3–4)	0.86 (2–4)	0.93
How to use the protocol?	0.93 (2–4)	0.93 (2–4)	0.93
Food consumption assessment	0.93 (1–4)	0.86 (1–4)	0.895
Flowchart	0.93 (2–4)	0.86 (1–4)	0.895
Recommendation 1 (R1): Encourage the daily consumption of beans”			
R1 - Advising	1 (3–4)	0.93 (2–4)	0.965
R1 - Suggestions for variations	1 (3–4)	0.93 (2–4)	0.965
R1 - Reason	1 (3–4)	0.93 (2–4)	0.965
R1 - Obstacles	1 (3–4)	0.93 (2–4)	0.965
Recommendation 2 (R2): Advise to avoid the consumption of sweetened beverages			
R2 - Advising	0.86 (1–4)	0.93 (1–4)	0.895
R2 - Suggestions for variations	0.86 (2–4)	0.86 (2–4)	0.86
R2 - Reason	0.93 (2–4)	0.86 (2–4)	0.895
R2 - Obstacles	1 (3–4)	0.79 (2–4)	0.895
Recommendation 3 (R3): Advise to avoid ultraprocessed foods.			
R3 - Advising	0.93 (1–4)	0.93 (1–4)	0.93
R3 - Suggestions for variations	0.93 (2–4)	0.86 (2–4)	0.895
R3 - Reason	1 (3–4)	1 (3–4)	1
R3 - Obstacles	1 (3–4)	1 (3–4)	1
Recommendation 4 (R4) - Advise the daily consumption of vegetables			
R4 - Advising	0.93 (1–4)	0.93 (1–4)	0.93
R4 - Suggestions for variations	0.86 (1–4)	0.93 (1–4)	0.895
R4 - Reason	1 (3–4)	1 (3–4)	1
R4 - Obstacles	0.79 (2–4)	0.93 (2–4)	0.86
Recommendation 5 (R5) - Advise the daily consumption of fruits			
R5 - Advising	1 (3–4)	1 (3–4)	1
R5 - Suggestions for variations	1 (3–4)	1 (3–4)	1
R5 - Reason	1 (3–4)	1 (3–4)	1
R5 - Obstacles	0.93 (1–4)	0.93 (1–4)	0.93
Recommendation 6 (R6) - Advise the user to eat in appropriate environments and with attention.			
R6 - Advising	0.93 (1–4)	0.93 (1–4)	0.93
R6 - Suggestions for changing habits	1 (3–4)	1 (3–4)	1
R6 - Reason	1 (3–4)	1 (3–4)	1
Additional Messages	0.86 (1–4)	0.79 (1–4)	0.825
Valuing the existing eating practice	0.93 (2–4)	1 (3–4)	0.965

Abbreviation: CVI, content validity index.

Note: The numbers in parentheses correspond to the minimum and maximum scores obtained in the clarity and relevance assessment using the 4-point scale: (1) “the item is not clear/relevant”; (2) “major revisions are required to make the item clear/relevant”; (3) “minor revisions are required to make the item clear/relevant”; and (4) “the item is clear/relevant”.

6.2–Face validity: Subsequently, the face validity of version 2 of the protocol was conducted, which aimed to identify the understanding and applicability of the protocol by its potential users – health professionals. Sixteen higher education-level health professionals who worked in PHC from the 5 regions of the country were invited to participate in online focus groups. The contributions from health professionals were considered to elaborate the final version of the protocol.

All focus groups were conducted by the team researchers, playing the roles of moderator and observer. Participants were instructed to keep the microphone off, activating it upon speaking. The desire to express an opinion was indicated by chat or by using the *raise your hand* feature, available on the virtual platform, and followed by the team members. All focus groups were recorded and later transcribed.

### Data Analysis of the Validity Step

For the analysis of the answers filled in the online form, the Content Validity Index (CVI), which measures the proportion of experts who expressed agreement regarding the clarity and relevance of each protocol component, was used. The CVI was calculated by the proportion of grades (3) and (4) divided by the total number of specialists, separated for clarity and relevance. The average CVI was also calculated considering a simple average of the CVI values for clarity and relevance. Components with CVI > 0.80 were considered adequate; that is, they did not need to be modified. 24

The justifications indicated in the online form were analyzed according to Bardin 25 in order to identify suggestions and comments related to the specificity of the pregnant woman. Based on the recognition of the most recurrent suggestions made by the specialists, two researchers defined themes for suggestions and selected demonstrative examples. Suggestions considered relevant were incorporated into version 2 of the protocol.

The transcripts of the focus groups with professionals were also evaluated with thematic content analysis. 25 Two team members read the transcripts, predefined the analysis categories, and extracted the related statements. The predefinition of categories and the extraction of the statements were monitored and verified by a third researcher of the team.

#### Ethical Aspects

All participants were voluntarily invited to participate in the study and signed a Free and Informed Consent Form. The focus groups were recorded and transcribed upon permission of the participants. The present study was approved by the ethics committee of the Universidade de São Paulo (4,232,862) and was conducted in partnership with the Ministry of Health.

## Results

The results of the steps described in the methodology will be presented in four topics: (1) Structuring the protocol, referring to steps 1 and 2 presented in the methods; (2) evidence on the dietary and nutrition needs of pregnant women, summarizing steps 3 and 4; (3) dietary advice messages for pregnant women, as a result of step 5; and (4) protocol validity, responding to step 6.

### Protocol Structuring

The adopted methodology resulted in a “Protocol of Use,” in compliance with the definition of the National Commission for the Incorporation of Technologies (CONITEC, in the Portuguese acronym) of the SUS, for dietary advice for pregnant women. The consumption assessment instrument that best met the protocol needs was the Food Consumption Markers form the Food and Nutrition Surveillance System (SISVAN, in the Portuguese acronym), since it is an instrument already implemented in PHC that addresses the main recommendations of the DGBP concisely and objectively through healthy and unhealthy eating markers.

The protocol was structured in the following subsections:

- Introduction: with brief information about the DGBP, characteristics of the pregnancy, and outline of the target audience of the nutritional advice, of the purpose of the protocol, and general instructions on the conduct when carrying out dietary advice.
- Step-by-step on how the protocol should be used.
- Instrument to assess food consumption: SISVAN food consumption markers.
- Decision flowchart: The flowchart directs the health professional to dietary advice according to the questions on the SISVAN form, to priority recommendations, and to the golden rule of the DGBP.
- Recommendations: Deals with dietary advice messages related to each consumption marker on the SISVAN form. Six recommendations were established.
- Valuing the existing eating practice: A message to encourage the continuity of healthy eating practices.
- Additional messages: Messages that were not covered by the SISVAN markers but are relevant in the dietary advice for the pregnant woman according to the literature review.

### Evidence on the Dietary and Nutrition Needs of Pregnant Women

The search in the databases found 24, 37, and 189 results in Scielo, Lilacs, and PubMed, respectively. Out of these, reading by title identified 57 articles eligible for abstract reading, excluding duplicates. After reading the abstracts, 17 manuscripts were excluded because they were not related to the purpose of the present review. Eventually, 35 scientific articles that investigated food consumption by pregnant women, key nutrients and nutritional demand, and the rapport between food and health outcomes were included for full reading. Additionally, information from technical materials, such as the “Pregnant Woman Booklet” published by the Ministry of Health, and original analyses of the 2017–2018 POF report, also supported the preparation of the protocol.

Evidence has shown that the consumption of a wide variety of in natura and minimally processed foods, as well as water, is particularly relevant to meet the need for essential nutrients for pregnant women, such as iron, folic acid, calcium, vitamin A and vitamin D, among others. According to data from the 2017-2018 POF, 23 in natura or minimally processed foods had a caloric share of 53% of the total calories consumed among pregnant women, with rice, beans, red meat, and poultry corresponding, jointly, to 29% of the calories in the diet of Brazilian pregnant women. Cooking ingredients, processed, and ultraprocessed foods, in turn, had an approximate caloric share of 16, 10, and 20%, respectively. 23

When comparing the food consumption patterns of pregnant and nonpregnant women between 18 and 38 years old, we could see a better diet in pregnant women. This is due to the greater caloric participation from in natura and minimally processed foods (1.82% higher in pregnant women compared with nonpregnant women), with emphasis on the consumption of fruit and offal items and the lower caloric participation of ultraprocessed foods in the diet of pregnant women (1.4% lower in pregnant women). Among ultraprocessed foods, there was a lower consumption of soft drinks and higher consumption of sausages and artificial fruit juice during pregnancy compared with nonpregnant women, leading to practices that require attention. 23

It is also noteworthy that, for a more adequate dietary advice, it is essential that the health professional is attentive to aspects related to social vulnerability and income, support network, the age of pregnant women, and their working conditions. Other factors that require attention in the gestational period are the physiological changes and symptoms that can influence food consumption in this period, such as nausea, vomiting and dizziness, heartburn, gastric fullness, constipation, weakness, fainting, physical pain, swelling, and oral changes. These symptoms may vary throughout pregnancy quarters and are manageable with adequate dietary advice for this period.

Finally, the process of extracting content from the DGBP identified 99 recommendations related to food consumption indicators evaluated by the SISVAN instrument and 5 additional recommendations considered relevant for inclusion in the protocol.

### Dietary Advice Messages for Pregnant Women

Based on the results presented in the previous topics, the dietary advice messages were developed contemplating six recommendations linked to the SISVAN markers: 1) encourage the daily consumption of beans; 2) advise avoiding the consumption of sweetened beverages; 3) advise avoiding the consumption of ultraprocessed foods; 4) encourage the daily consumption of vegetables; 5) encourage the daily consumption of fruits; and 6) encourage the user to eat in appropriate environments and with attention. Each recommendation addresses the dietary advice that should be offered, suggestions and healthy alternatives, justification that supports the professional about the advice, possible obstacles that can make it difficult to adhere to the advice, and strategies to overcome them. Furthermore, additional messages were elaborated for important issues regarding the diet pregnant women not covered by the SISVAN markers. Example of the elaborated messages can be found in Chart 1 .

**Chart 1:** Example of messages elaborated for dietary advice for pregnant women

SISVAN form question	Individual's answer	Dimension of recommendation	Dietary advice messages
Did the user consume sweetened beverages yesterday?	No	Value the existing eating practice	“Value the healthy eating practice as it strengthens the practice, encourages its continuity, and can provide important information around healthy diets. You may use the justification to value the existing eating practice.”
	Yes	General recommendation	“Recommend pregnant women to avoid the consumption of sugar-sweetened beverages such as soft drinks and artificial juices, which are also known as ultraprocessed beverages.”
		Suggestion on healthy alternatives	“Encourage the consumption throughout the day of pure water that is proper for human consumption, or 'enriched' with slices of lime, mint leaves, or pineapple peel. In case of nausea, encourage the consumption of cold water”.
		Reason	“Sweetened beverages are not recommended for pregnant women, as they are usually added with a lot of sugar, flavorings, dyes, and other cosmetic additives, in addition to many of them having caffeine in the composition. Such compounds can worsen common symptoms of pregnancy, such as nausea and vomiting, and, if they contain caffeine, can increase the risk of miscarriages, premature births, low birthweight, and stillbirth. The consumption of these drinks can also interfere with the water intake, which, during this period, is especially important to boost blood circulation and irrigation of the uterus and the placenta, to maintain amniotic fluid at adequate levels, to stabilize blood pressure, in addition to eliminating toxins that increase the risk of urinary infections”.
		Understanding and overcoming obstacles	“For pregnant women who do not frequently drink much water, advise them to carry bottles of water in their bags when leaving home, ensuring access to drinking water during the day, in addition to saving money. At home or at work, always keep a bottle of water at hand.”
**Additional messages**			
"Sweeteners are not recommended during pregnancy, as they may increase the likelihood of premature birth and of development of asthma in children up to 7 years old, in addition to being associated with metabolic changes in women and with a higher likelihood of overweight in children in early childhood."			

**Source:** Ministério da Saúde and Universidade de São Paulo (2021). 34

### Protocol Validity

Version 1 of the protocol, submitted to content validity by a panel of experts, had 14 female participants, from the South ( *n =* 3), Southeast ( *n =* 8), Midwest ( *n =* 1), and Northeast ( *n =* 2) regions of the country, with higher education in nutrition ( *n =* 11), nursing ( *n =* 2), or medicine ( *n =* 1), and working in the academic and research, public management, assistance, or primary healthcare areas. The panel took place in November 2020. All protocol components had an average CVI above the cutoff point (0.80). Even so, all contributions from experts in each component were analyzed individually. Out of the 29 components evaluated, 14 obtained total agreement (CVI = 1.0) for clarity and 09 for relevance ( Table 1 ). According to the assessors, the components that most required changes were recommendations 2 and 4 as well as additional recommendations.

Some suggestions made by the specialists in the components that required more attention according to the results of the CVI analysis and that were incorporated into the protocol are presented in Chart 2 . In addition to suggestions for changes and comments on relevance and clarity, the experts brought elements about signs/symptoms and dietary strategies to be considered for pregnant women, specific nutritional needs, health outcomes, need for comprehensive care by health professionals, food safety, and broadening the look at issues related to eating habits and the regional diversity of the diets of pregnant women.

**Chart 2:** Suggestion theme and its definition, example of suggestion and modification in the Protocols for the Dietary Guidelines for the Brazilian Population use in nutrition advising for pregnant women

Suggestion theme and definition	Suggestion example	Example of change made to the protocol
***Clarity*** Suggestions related to the adequacy of technical terms, to the understanding of meanings and information, and technical content qualification.	“Specify the size of the coffee cup that can be consumed.”	*Regarding caffeine consumption, a safe intake should not exceed 100 mg of caffeine, which is equivalent to a cup of caffeinated coffee or tea (such as mate tea, black and green teas, etc.).*
***Specific needs*** Suggestions related to nutritional needs and specific recommendations for pregnant women.	“Regarding specifically liver, the professional needs to know what to recommend. It is common that its consumption is encouraged during pregnancy. Should this be done? what is a safe weekly consumption without risk of excess vitamin A?”	Offal, such as liver, is food source of iron and vitamin A, an essential nutrient in this period. If the pregnant woman is used to eating that, remind her of the importance of including it in a meal along the week.
***Health outcomes*** Suggestions regarding the most common health outcomes (weight gain, gestational diabetes, and hypertension).	"Warn about excessive weight gain especially in ultraprocessed and sweetened drinks advice".	Greater consumption of ultraprocessed foods during pregnancy is related to excessive weekly weight gain and greater chances of postpartum weight retention, factors that are related to negative maternal and child health outcomes, such as the development of obesity in the mother, and higher birthweight in infants.
***Food safety*** Suggestions related to hygiene and food handling relevant to pregnant women.	“I suggest incorporating advice on the relevance of the correct hygiene of vegetables that will be consumed raw”.	Many people may not consume fresh foods for fear of contamination by toxoplasmosis. Advise them to wash the fruits or vegetables under running water and then place them in a solution of 1 spoon of sodium hypochlorite or 1 spoon of bleach suitable for using in food to a liter of water for 15 minutes. After this period, rinse in running water. Food will thus be ready and safe for consumption.

The face validity with health professionals, carried out with version 2 of the protocol, had the participation of 6 professionals, mostly women ( *n =* 4), working in PHC in the south ( *n =* 1), southeast ( *n =* 4), and northeast (1) regions of Brazil, including a nutritionist ( *n =* 1), doctors ( *n =* 2), and nurses ( *n =* 3), and took place in December 2020. The thematic analysis of the focus group transcripts identified two major themes and their respective categories and subcategories of analysis. The theme "technology in health" covered presentations that demonstrate the ability of the protocol to qualify and update health professionals for dietary advice for pregnant women based on the best technical and scientific evidence, in addition to contributing to promote the confidence of professionals to address the healthy eating issue. The “applicability” theme, on the other hand, gathered reports that point out possible challenges related to the work process with potential influence on the applicability of the protocol in prenatal appointments, while indicating pregnancy as an opportune phase for diet changes and subsequent application of the protocol. The themes and their respective categories and subcategories are presented in Table 1 with examples ( Chart 3 ).

**Chart 3:** Themes, categories and subcategories found in the face validity with health professionals in relation to the Protocol for the Dietary Guidelines for the Brazilian Population use in nutrition advising for pregnant woman

Topic: Health technology
**Category: Qualification of dietary advice**
*Subcategory 1: Qualification of dietary advice when handling common pregnancy signs and symptoms:* Example: *“...* food is the biggest challenge. Because when she arrives to start prenatal care at the unit, what is her main complaint? Nausea, vomiting, difficulty in eating... these are the advice that I use to not focus on. We do use to focus on other things and forget about it a little... I myself have a hard time saying: oh, what food items can you avoid? You talk a little about coffee. Which I found amazing. And that was something I didn't even talk to them about, which I now intend to start talking about. And…but which food items can they avoid that will cause less nausea? Which food items cause flatulence the most, right? They have a lot of questions about it. And we used to approach it in a very generic way: 'Ah, avoid sweets, you know, avoid saturated fats that are deep-fried foods. Avoid excess salt. These foods that are processed, right…”
*Subcategory 2: Qualification of dietary advice by other categories of health professionals (in addition to nutritionists):* Example: “… so, here in my job, for the pregnant woman to get to an individual appointment with the nutritionist, usually faces some changes already… [such as] excessive weight gain, some pathology, or a high-risk prenatal care. In addition, we make group approaches and who will provide this service are the doctors, nurses, and nursing technicians. In addition, we make group approaches and who will provide this service are the doctors, nurses, and nursing technicians. And many times, … in my practice, with the rush, diet goes a little unnoticed.”
*Subcategory 3: Qualification of dietary advice for pregnant women based on scientific evidence and the food culture of Brazilian people:* Example: “... you talked in the leaflet a little, you know, about social classes, right, and about consumption, you know, that white women, white pregnant women usually consume a little more... not a little, right... more healthy foods. The mulatto and black women, yes, consume more industrialized foods, right, processed. And for me, this is essential. Very important. So yeah, I wish I had material on that, because I can't find it, I can't get it. It is not accessible. Most of the women that I start prenatal care today, in my region, right, I work in the south region, extreme south, are mostly black and mulatto women. So, this woman is the woman who consumes more ultraprocessed foods… but for me it is essential that I look at this data that exists about black and mulatto women, who consume more ultraprocessed foods, and that I can advise them in an accessible way, the foods that are also available in other ways and that are healthier for them during this time…”
*Subcategory 4: Qualification of dietary advice for pregnant women based on the recommendations of the Dietary Guidelines for the Brazilian Population:* Example: “... going back to the case of pregnant women, I usually use the Guideline very little, because it has been very comprehensive. And maybe, it is, to think of a protocol, in specific guidelines, for pregnant women. For me, it's very interesting.”
**Category: Promoting the trust of professionals** Example: “So, yeah, for me, yes, it gives me a lot more confidence. I realize that, over time, as we are in a very dynamic job, there is a lot going on, right, no one was expecting, for example, this pandemic, and there are guidelines all the time, every day we receive something new; then I feel that, during my practice, many things end up fading into oblivion, or we end up losing a little, right. So, I want material that makes me think always, yes, that doesn't let me fade into oblivion, right, that makes me confident so that I can always offer this protocol, right, and that is always relevant. Of course, things are changing all the time, but with the material that I can, that I can offer that and I think it's going to offer me a lot more confidence, for sure.”
**Topic: Applicability**
**Category: Challenges and spaces for using the protocol in prenatal appointments**
*Subcategory 1: Challenges linked to the health work process that influence the applicability of the protocol:* Example: “... yes, what I think will be a great challenge, J., at least in my job, is that I still see professionals who are not nutritionists, with difficulty, … the routine of fitting in the filling of the food consumption marker, which is the starting point for the protocol. The protocol, it will be based on you filling in the marker and from the marker responses, you should guide that. Yeah, and then I think that, I don't know, it's up to us to think of strategies to encourage this filling, if not in all appointments or for everybody, yes, the entire population served, but focused, at least in this period, to be able to provide advice for pregnant women. So, kind of, first, it would be important for us to understand food as a health determinant and that it belongs to all of us and how important it is to be addressed. And the marker is a kickoff. So, I think this is a big initial challenge.”
*Subcategory 2: Pregnancy as an opportune phase for changes in diet and subsequent application of the protocol.* *Example:* “... and especially the pregnant women, right, which are a group that I see like that, in my job, that is looking for care, self-care, is looking for more information. It's a group that's more interested, mainly, in nutritional information, right, in nutrient information, vitamins, and everything else, and then, I think the more knowledge we have, the simpler, both for us to learn and to pass on to these pregnant women, I think it will flow much better and it is very helpful. Because we have a lot of extensive material, a lot of long stuff and we can't stop to read it. And we end up losing super useful knowledge, but that we can't read. You can't stop to acquire that knowledge.”

## Discussion

The present study describes the process of elaborating and validating a protocol for the use of the DGBP in dietary advice for pregnant women in individual appointments. The protocol elaboration steps included the formation of a technical team, definition of the protocol format, definition of the food consumption assessment instrument, extraction of recommendations from the DGBP, literature review related to the dietary needs of pregnant women, development of advice messages and, finally, content and face validity of the protocol. The product resulting from these steps was well evaluated by specialists in food, nutrition, care, and women's health, as well as by the target audience, proving to be clear and relevant for use in the clinical practice of professionals working in PHC.

Although the importance of adequate nutrition during pregnancy is known, to the best of the authors knowledge, there are no studies to date that developed recommendations on healthy eating during pregnancy aimed at health professionals, guided by theoretical-scientific references, applicable throughout the country, and comprising different regional and socioeconomic contexts. Nutritional advice during pregnancy nationwide is only found in the Pregnant Woman Booklet, in the form of “10 steps to a healthy diet.” 26 However, this instrument is aimed at pregnant women, with general information about nutrition during pregnancy, and it is not possible to identify which diet issues need to be prioritized.

In Brazil, other publications are found in the academic literature, such as the validation of an educational booklet for healthy eating during pregnancy with regional foods 27 and the elaboration and evaluation of educational material for pregnant women in the northeast region of the country on the importance of adequate diet during pregnancy, with emphasis on vitamin D. 28 However, the first publication adopts a technical reference on nutrition that is already outdated, and both publications use a language that addresses the pregnant woman as a target audience. In addition, although significant, they deal with local efforts and, in general, they were not developed with the same objectives as the instrument presented in the present publication, limiting their comparability.

Studies that propose to develop protocols to guide clinical practice in pregnant women care usually have a nutrient-centered approach or focus on preventing excessive gestational weight gain. 14 29 In the second case, the purpose of these propositions considers, above all, the relevance of this prognosis in negative outcomes in the health of the mother and of the child. The protocol proposed in the present study advances by proposing an instrument that, by recognizing the DGBP as a theoretical-scientific reference, focuses both on the promotion of healthy eating and on the prevention of excessive weight gain. This occurs because the Brazilian Guidelines adopt a multidimensional paradigm of healthy eating, including cultural, biological, economic, and environmental aspects, in addition to considering the level of food processing. This approach includes robust evidence on the rapport between healthy eating and positive health outcomes and between consumption of ultraprocessed foods and excessive weight gain during pregnancy, as well as postpartum weight retention, and other negative maternal and child health outcomes, such as development of maternal obesity, and higher birth weight in infants. 13 30 31 In this sense, the protocol, in addition to guaranteeing recommendations on healthy eating for pregnant women who are not at nutritional risk, also favors those who present some risk of excessive weight gain, allowing its use in the range of demands of individual care for pregnant women.

Another relevant feature of the protocol is its potential for use in in-service training initiatives to qualify the workforce in food and nutrition. The need to invest in the training of health professionals to promote healthy eating is recognized, in view of the global emergence of preventable chronic noncommunicable diseases through healthy eating. 32 The developed protocol was satisfactorily identified in the evaluation panels with professionals as a health technology that focuses on the qualification of professional practice. They recognized the potential of the instrument to assist in the clinical management of pregnant women, in addition to feeling more confident to advise on food, especially those who are not nutritionists, as they are using a tool based on a theoretical framework, giving value to the DGBP and implementing it in professional practice.

Although the importance of following methodological steps in the development of clinical protocols is consolidated, few studies elucidate in detail the process of building protocols in the dietary guidelines for pregnant women. 21 The protocol presented in the present study was developed following rigorous methodological steps of development and evaluation proposed by guidelines, enabling its reproducibility. 8 20 21

Although the validation performed by a panel of experts is the most used to validate protocols in the health area, 33 the process carried out in the present study may have some limitations. The difficulties posed by the COVID-19 pandemic required adaptations in the methodology, such as carrying out the evaluation panels virtually. This format may have limited the interaction between participants and hampered the discussion. In addition, there was difficulty for health professionals to participate due to the work overload on the front line and the unavailability for prior reading of the material and participation in the panels. As a way of trying to overcome these obstacles, the meetings were held after working hours and with reduced time.

Finally, there is a need for additional efforts in the dissemination and implementation of the material among health professionals working in PHC, promotion of nutrition training in the permanent health education agenda, and maintenance of the routine service in the use of the tool. In the focus groups carried out in the present study, professionals pointed out the potential for protocol inclusion in prenatal appointments. The protocol was published by the Ministry of Health in 2021 34 and is available for consultation on the website of the virtual health library (available at: https://bvsms.saude.gov.br/bvs/publicacoes/protocolos_guia_alimentar_fasciculo3.pdf ). Future works will evaluate the effectiveness of an educational intervention for the use of the protocol in the qualification of the practice of health professionals.

## Conclusion

The developed and validated instrument presented in the present study sought to fill an important gap in the development of clinical protocols for individual dietary advice for pregnant women. In addition, the protocol seems promising to qualify the performance of health professionals working in PHC and enables the implementation of the DGBP in clinical practice in individual care.
